# Supplementary material for: Turbulent coherent structures and early life below the Kolmogorov scale
Source: Nat Commun. 2020 May 4;11:2192. doi: 10.1038/s41467-020-15780-1 (PMC7198613; doi:10.1038/s41467-020-15780-1)
Supplement: Supplementary file 1 — Supplementary Information [file 41467_2020_15780_MOESM1_ESM.pdf]

SUPPLEMENTARY INFORMATION: Turbulent coherent structures and early life below the Kolmogorov scale

Madison S. Krieger, Sam Sinai, and Martin A. Nowak

Supplementary Note 1: Life at low Damköhler number

To vary the Damköhler number, either the biological rates must be scaled up or down or the representative timescale of the fluid must change. We opted for the latter, since all relevant fluid timescales scale linearly in Γ_i, the strength of the individual point vortices. In all other sections, we had $|\Gamma| = 1$; for this section, we employed $|\Gamma| = 0.001$ (**Supplementary Movie 1**) and $|\Gamma| = 100$ (**Supplementary Movie 2** and Fig. 2).

Figure 2 was generated by simulating passive tracers in N=7 vortices with $|\Gamma| = 100$ from $t = 0$ to $t = 20$ $1,000$ times and analyzing the components of the geometric graph induced by $R_{int} = 0.03$.

Supplementary Note 2 - A further assessment of the role of diffusion

Here we briefly revisit our decision to neglect the role of thermal diffusion in our modeling, using distance and number statistics from our simulations.

We note that, as a consequence of our decision to model particles in a turbulent flow, in certain limits our values of the Damköhler number Da employed must be interpreted in only one of the two possible ways. For instance, high Damköhler number could be interpreted theoretically as either very quiescent flow or very fast replicators. However, a very quiescent flow cannot be expected to be turbulent. As a consequence, no coherent structures would form. Our argument that, in a turbulent flow, the Batchelor scale (the relevant lengthscale for thermal diffusion) is orders of magnitude smaller than the Kolmogorov scale would become moot in the absence of turbulence. Therefore, if high Da is interpreted to mean that the flow is quiescent, we would indeed be making large errors in neglecting diffusion. In all limits of Da, then, we assume the interpretation in which the fluid remains in motion.

When this is the case, it is still true that on the lifetime of a coherent structure, diffusion plays a rather negligible role. However, the lifetime of a coherent structure is only a useful timescale if the Damköhler number is not very small. In this limit, particles survive for extremely long times, and therefore the small perturbations due to diffusion could potentially add up. We expect this to be important when (a) there are only a few particles interacting at any given time, and (b) they are interacting at distances where we would be concerned that the neglected diffusive forces would have taken them out of the range of interaction, and therefore we are over-counting catalytic actions.

As seen in Fig. 3, (as well as Fig. 2), this is true when the Damköhler number is very small. However, in these regimes we also never saw establishment, due to the observation in Fig. 2 that interactions are simply too infrequent in this domain. Since our system is explicitly calibrated such that the particles die out with high probability when there are no interactions, this is already an extremely deleterious regime for the emergence of cooperative metabolisms. Already at $Da = 0.01$, we had on average around 21 particles in clusters of maximum span comparable to R_{int}. Here, already, errors due to neglecting diffusion seem minimal, and decrease in size with increasing Da (subject of course to our above caveat that high Da must mean faster replication).

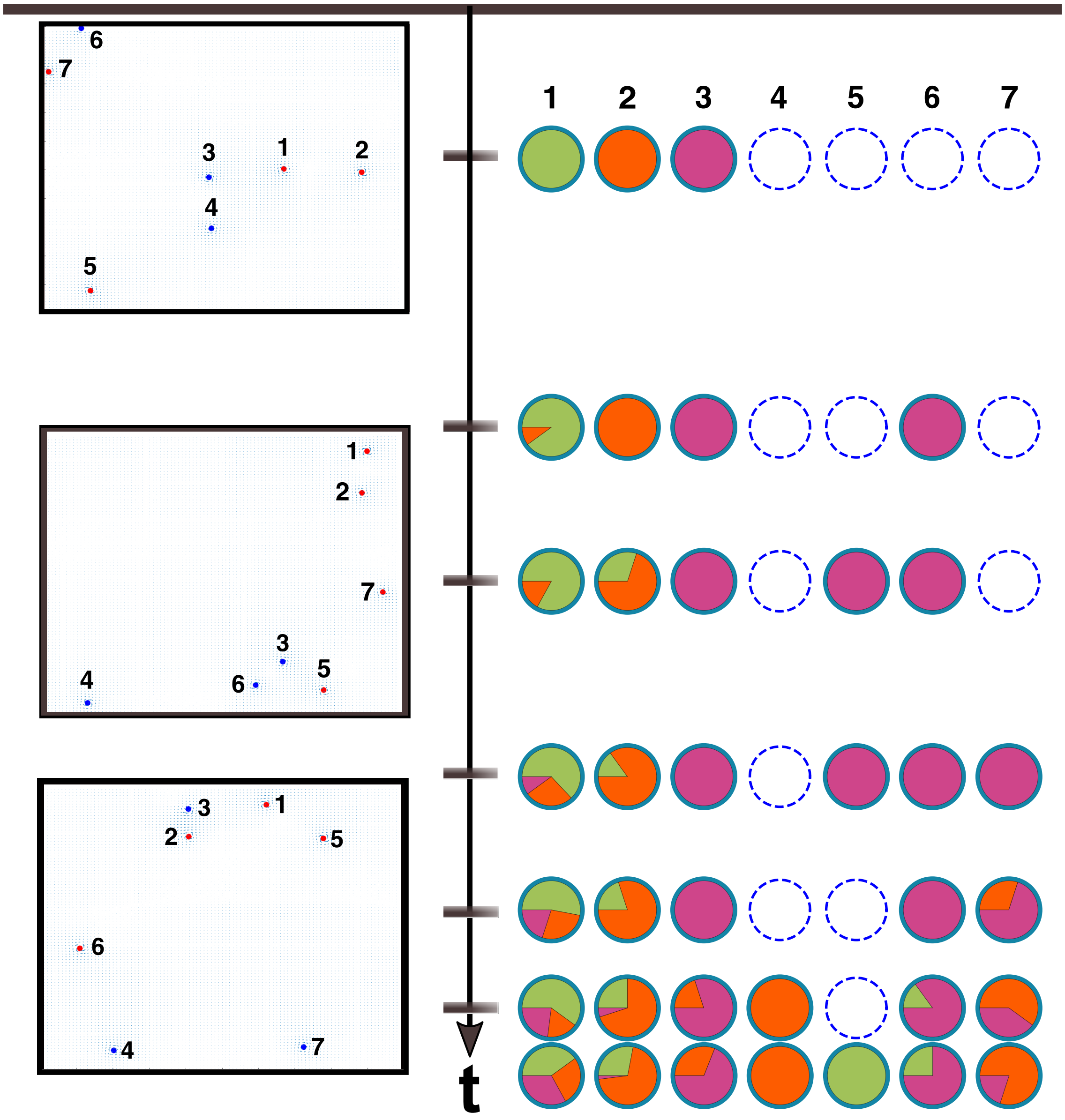

Supplementary Figure 1. Migration leads to genetic diversity. In addition to the concept of LCSs splitting and merging (which also promote genetic diversity), we also observed "migration" events in which a handful particles were cleaved from a parent vortex and traveled quickly along a high-FTLE ridge to a new vortex or transient coherent structure. This mechanism proved especially effective at combining genetic material from different vortices as they passed by one another, for instance in this realization of a seven-vortex system supporting a 3-species hypercycle. We initialized three "flourescently dyed" lineages in three parent vortices at $t = 0$ and observed the spread of these lineages (shown here, the fraction of the disk in a particular color represents the total fraction of all particles from a particular lineage) over time. Drawn here is one realization demonstrating founder-like effects, in which the first migrating particles to reach a new vortex tend to occupy a large fraction of the structure for long times.

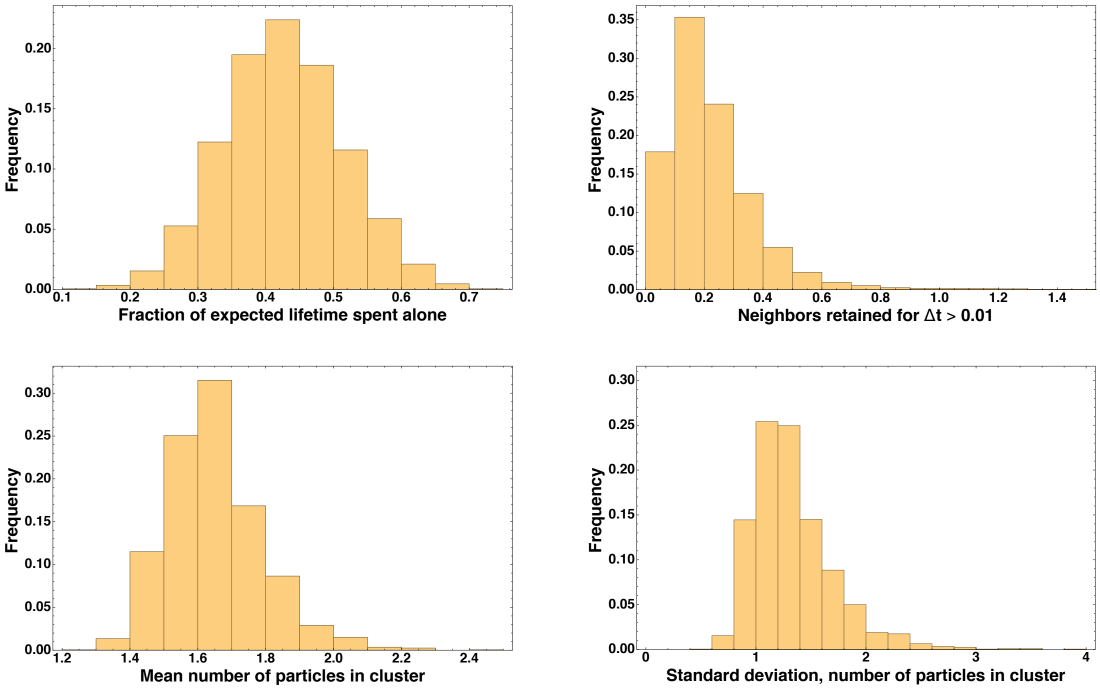

Supplementary Figure 2. Very low Damköhler flows make cooperation challenging. While decreasing the Damköhler number greatly increases the cost of simulations on replicating populations, we can get an idea of the challenges to cooperation in this limit by considering the statistics for a population of 500 passive tracers in a thousand simulations. (Top-left) The average time that a passive particle spends at a distance greater than $R_{int} = 0.03$ from any other particle divided by the expected particle lifetime ($t = 1$). (Top-right) The likelihood of retaining a particular neighbor over one timestep in our simulations ($\Delta t = 0.01$). (Bottom-left) The mean number of particles in a "cluster" — in a given snapshot of the flow, most particles are isolated from one another. (Bottom-right) The standard deviation in cluster size.

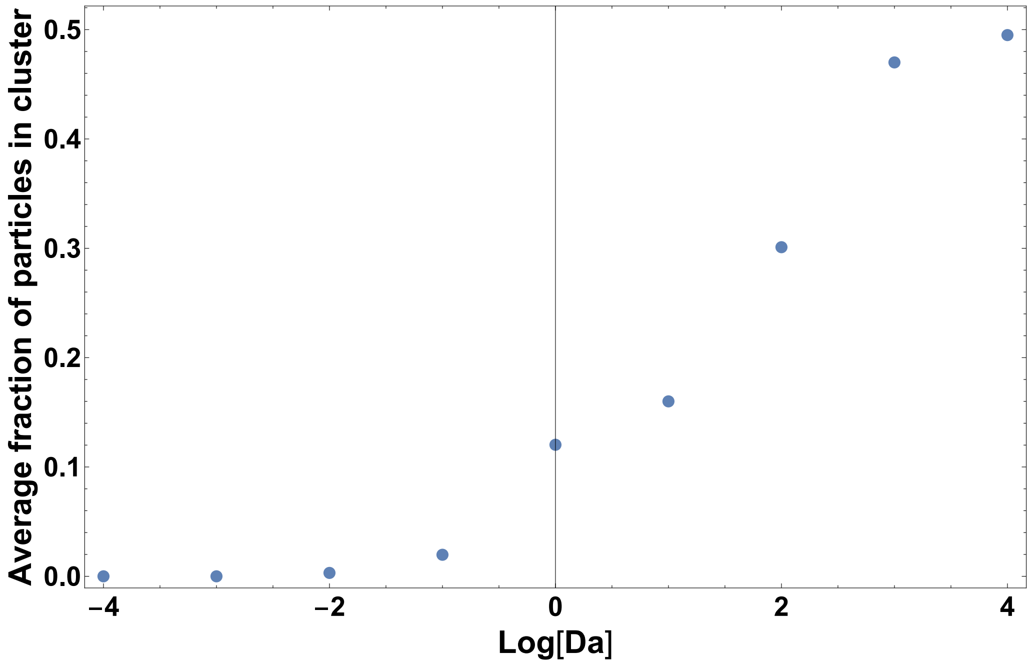

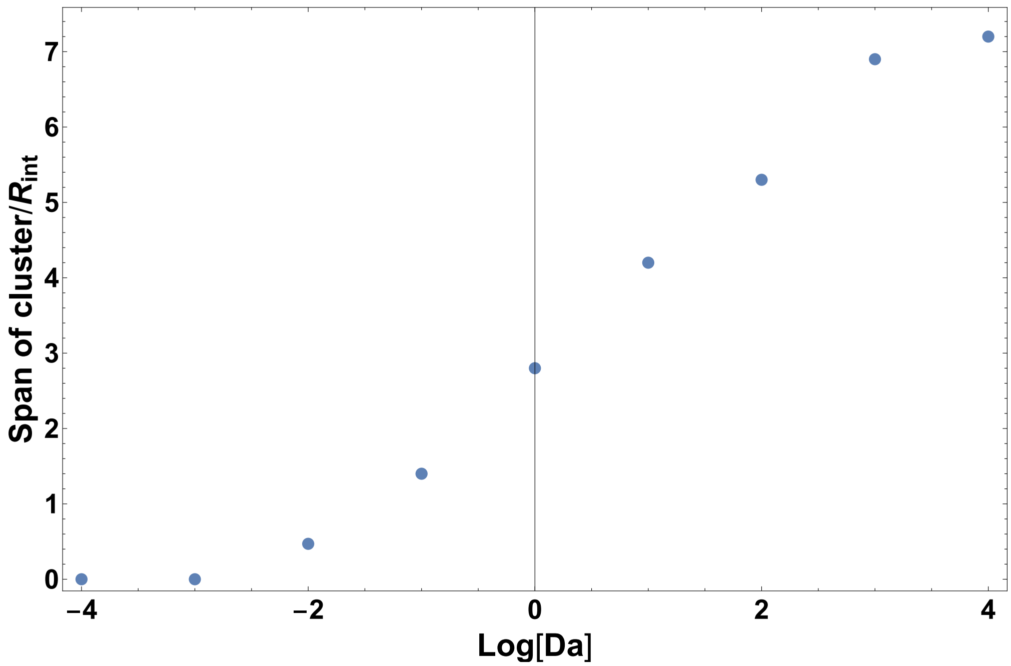

Supplementary Figure 3. Assessing the range of importance of thermal diffusion. Top: At the end of each "establishment" R2 simulation as a function of Damköhler numbers, we counted for each cluster of particles the total fraction of all established particles that were in that cluster. The larger the size, the less likely the impact of diffusions across the Batchelor scale. **Bottom:** For the same data set, we measured the spatial span (defined as the maximum distance between any two particles that were in the same cluster). When this span is small relative to the maximum distance of an interaction, R_{int}, then diffusion is more important since neglecting it could lead to over-counting of interactions. Note that in both panels: (a) establishment was never seen below $Da = 0.01$, (b) establishment simulations end at $2,000$ particles, and (c) in these simulations $R_{int}/L = 0.03$. Diffusion is seen to be more important at very low Damköhler numbers; however, here establishment is already rarely seen in our simulations.
