# Supplementary material for: Turbulent coherent structures and early life below the Kolmogorov scale
Source: Nat Commun. 2020 May 4;11:2192. doi: 10.1038/s41467-020-15780-1 (PMC7198613; doi:10.1038/s41467-020-15780-1)
Supplement: Supplementary file 3 — Description of Additional Supplementary Files [file 41467_2020_15780_MOESM3_ESM.pdf]

Description of Additional Supplementary Files

File Name: Supplementary Movie 1
Description: A realization of the “Replicase R2” metabolism in a flow at high Damkoehler number (Da=1,000).

File Name: Supplementary Movie 2
Description: A realization of the “Replicase R2” metabolism in a flow at low Damkoehler number (Da=0.001).

File Name: Supplementary Movie 3
Description: A realization of the “Replicase R2” metabolism in a flow at Da=1.
